# The role of the lymphatic system in cholesterol transport

**DOI:** 10.3389/fphar.2015.00182

**Published:** 2015-09-02

**Authors:** Li-Hao Huang, Andrew Elvington, Gwendalyn J. Randolph

**Affiliations:** Department of Pathology and Immunology, Washington University School of Medicine, St. Louis, MO, USA

**Keywords:** lymphatics, cholesterol reverse transport, cholesterol efflux, atherosclerosis, high-density lipoprotein transport, high-density lipoprotein modification, interstitial space, apolipoprotein A1

## Abstract

Reverse cholesterol transport (RCT) is the pathway for removal of peripheral tissue cholesterol and involves transport of cholesterol back to liver for excretion, starting from cellular cholesterol efflux facilitated by lipid-free apolipoprotein A1 (ApoA1) or other lipidated high-density lipoprotein (HDL) particles within the interstitial space. Extracellular cholesterol then is picked up and transported through the lymphatic vasculature before entering into bloodstream. There is increasing evidence supporting a role for enhanced macrophage cholesterol efflux and RCT in ameliorating atherosclerosis, and recent data suggest that these processes may serve as better diagnostic biomarkers than plasma HDL levels. Hence, it is important to better understand the processes governing ApoA1 and HDL influx into peripheral tissues from the bloodstream, modification and facilitation of cellular cholesterol removal within the interstitial space, and transport through the lymphatic vasculature. New findings will complement therapeutic strategies for the treatment of atherosclerotic vascular disease.

## Introduction

**FIGURE 1:**
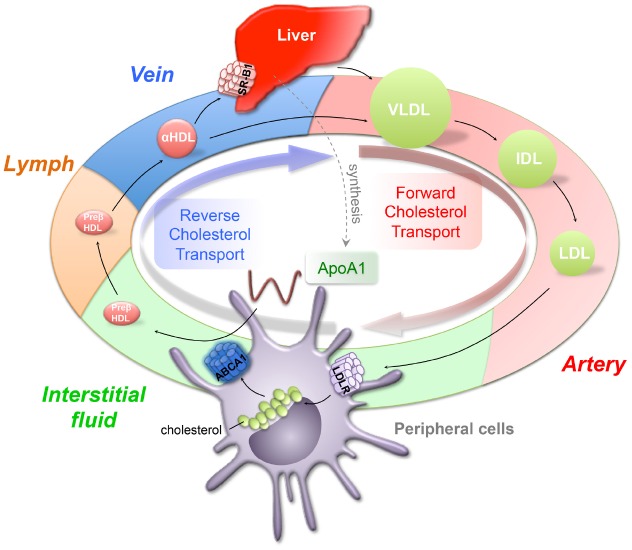
**Forward and reverse cholesterol transport.** The forward cholesterol transport starts from the liver, which generates triglyceride-rich verylow-density lipoprotein (VLDL) and releases it into circulation. VLDL is progressively hydrolyzed and forms intermediate and low-density lipoproteins (IDL, LDL). LDL, as a major cholesterol carrier, serves to transport cholesterol to peripheral tissues to use through LDL receptor (LDLR). To remove the excess accumulated cholesterol from peripheral tissues, cholesterol is transported through lymphatics, veins, and back to the liver for excretion, a process called reverse cholesterol transport (RCT). The high-density lipoprotein (HDL) plays an important role in removing cholesterol through RCT. Lipid free ApoA1 picks up free cholesterol from peripheral cells through ATP binding cassette transporter A1 (ABCA1) and forms preβ-HDL. Preβ-HDL travels through lymphatics to the vein, where preβ-HDL can be further modified to become α-HDL and removed through direct uptake by HDL receptor, scavenger receptor B1 (SR-B1).

Lipoproteins are important vehicles to transport hydrophobic lipids in the circulation of the human body. They contain various lipids complexed with specific apolipoproteins, which function as a ligand for transporters expressed in tissues and to maintain the structural properties of the lipoprotein particles. Lipoproteins can be distinguished based on their particle size and density. Forward cholesterol transport starts from very low-density lipoproteins (VLDLs), which mainly contain triglycerides and a small amount of cholesteryl esters and free cholesterol. They are exported from the liver and delivered to peripheral tissues for use (Figure 1). After removal of triglyceride content by lipoprotein lipases, VLDLs are converted to smaller intermediate-density lipoproteins (IDLs) and low-density lipoproteins (LDLs). LDLs account for the majority of cholesterol delivered to peripheral tissue for usage (Figure 1; Hewing and Landmesser, 2015). In order to remove excess cholesterol content from peripheral tissues, cellular cholesterol is removed, passed to the liver and packaged in bile salts for ultimate excretion, a process called reverse cholesterol transport (RCT; Figure 1). The initial step in RCT is the efflux of cellular cholesterol, mediated by ATP-binding cassette (ABC) transporters expressed on the cell, to complex with apolipoprotein A1 (ApoA1) to form nascent high-density lipoprotein (HDL) particles. This initial step occurs within the interstitial space, where nutrients and waste products are exchanged between cells and capillaries (Figure 1). The composition and properties of the interstitial fluid varies between tissues and is modulated in response to physiological and pathophysiological conditions (Hansen et al., 2015). Dating back to mid-1970s, an inverse relationship was reported between the plasma concentration of HDL cholesterol and the risk of a detrimental cardiovascular event. Unfortunately, strategies that effectively raise plasma HDL levels, such as cholesteryl ester transfer protein (CETP) inhibitors and niacin, have not reduced clinical cardiovascular events. Despite experimental evidence that HDL particles function in an anti-atherosclerotic manner (Plump et al., 1994; Rosenson et al., 2012), clinical studies have failed to demonstrate that plasma HDL concentrations are directly correlated with and regulate atherogenesis.

We propose that in order to better understand why elevating plasma HDL concentrations do not always comprise an efficacious cardiovascular disease (CVD) therapy, one must understand the different compartments in which HDL localizes. HDL concentration, composition, and modification is different in the interstitial space as compared to plasma HDL. Miller et al. (1976) theorized in 1976 that the lymphatic vasculature could be the main transit route for movement of HDL from the interstitial space to the bloodstream and liver. Experimental evidence supports this suggestion: surgical or genetic disruption of lymphatic vasculature in the skin of mice markedly reduces the appearance of labeled cholesterol in plasma that originated from implanted tissue macrophages (Lim et al., 2013; Martel et al., 2013). Therefore, a better understanding of how HDL is formed, how it gains access to peripheral tissues, how it is modified in the interstitial space and in lymphatic vessels, and finally is transported through lymphatics to the liver for excretion is essential to the development of novel HDL-based therapeutics to prevent or ameliorate CVD. This review discusses current clinical findings related to RCT, HDL transport and modification in the interstitial space, and the role of the lymphatic system in RCT, and identifies potentially important questions for future research.

## The HDL Hypothesis Needs Revision

Many human epidemiological studies demonstrate a strong inverse association of plasma levels of HDL cholesterol and coronary heart disease (CHD), which suggest that raising HDL cholesterol levels may decrease risk of CVD (Barter et al., 2007a; Singh et al., 2007). Independent of LDL concentration, a 1% increase in the concentration of HDL cholesterol reduced CHD events 0.6% (Gordon et al., 1986). In the Helsinki Heart Study, an increase in HDL cholesterol, together with a decrease in LDL cholesterol concentration, was significantly associated with decreased in CHD events, independent of other risk factors (Manninen et al., 1988). While promising, several recent clinical trials aiming to increase HDL cholesterol levels failed to support these early findings. In randomized trials, despite significantly increased HDL cholesterol in the plasma, treatment with niacin or CETP inhibitors did not improve cardiovascular outcomes (Barter et al., 2007b; AIM-HIGH Investigators et al., 2011; Schwartz et al., 2012; HPS2-THRIVE Collaborative Group et al., 2014; Keene et al., 2014). One can argue that the effects of niacin were confounded by concurrent statin treatment and that taking niacin alone would be associated with a decreased rate of non-fatal myocardial infarction. Even so, niacin itself also decreases LDL cholesterol so that reduced non-fatal myocardial infarction events might be simply interpreted as being due to reduced LDL (Keene et al., 2014). Thus, trials using pharmaceutical interventions to test the HDL hypothesis have caveats, and the conclusions remain unclear. A more specific strategy of using ApoA1/HDL mimetics was developed and used to test HDL cholesterol effects on CVD (Kingwell and Chapman, 2013). Unfortunately, a phase 2b randomized trial with the HDL-mimetic CER-001 demonstrated no significant difference in plaque regression between treated and non-treated groups (Tardif et al., 2014). Human genetic studies showed that genes regulating cholesterol levels are poorly correlated with protection from vascular diseases (Voight et al., 2012). Another human genetic study showed that an endothelial lipase variant, N396S, raised HDL cholesterol levels, but was not associated with decreased CVD (Voight et al., 2012). Inversely, the ApoA1 milano mutation, which creates a disulfide bond in the c-terminus facilitating increased lipid recruitment relative to WT ApoA1 (Gursky et al., 2013), does not lead to any increase in CVD even though carriers display very low plasma HDL cholesterol levels (Favari et al., 2007).

## Cellular Cholesterol Efflux and Cardiovascular Diseases

The consequences of dysfunctional cellular cholesterol efflux are best observed in the context of the pathophysiology of CVDs. Khera et al. (2011) reported that *in vitro* cellular cholesterol efflux capacity, assessed by an assay originally developed by Rothblat et al. (1999), was strongly inversely associated with CVD, consistent with the experimental outcomes demonstrating HDL is atheroprotective by facilitating cholesterol efflux from foamy macrophages. The authors conclude that cellular cholesterol efflux capacity is a more accurate biomarker for CVD than plasma HDL cholesterol levels (Khera et al., 2011). Furthermore, Rohatgi et al. (2014) showed that cholesterol efflux capacity was inversely associated with CVD after adjusting for the traditional risk factors, HDL cholesterol levels, and particle concentration, suggesting that cholesterol efflux capacity is a new biomarker for CVDs. Paradoxically, Li et al. (2013) observed that higher cholesterol efflux capacity correlated with increased morbidity and mortality of incident CVD, such as myocardial infarction and stroke. In line with this finding, metabolic diseases that are risk factors for CVD, such as type IV hyper-triglyceridemic and Type II diabetes, also showed higher cholesterol efflux from foamy macrophages through the ABC transporter A1 (ABCA1) dependent pathway (Attia et al., 2008), likely due to elevated triglycerides (Yassine et al., 2014). One speculative but attractive explanation for the puzzling results by Li et al. (2013) is that although the individuals under study had high plasma HDL, they also bore physiological alterations that led to poor cycling of HDL (Randolph and Miller, 2014). In this scenario, plasma HDL would appear quite functional *in vitro*, but *in vivo* it may not be located or functional in the extravascular spaces where its action is required, and genome-wide association study (GWAS) finds that HDL transport pathway is associated with coronary artery disease patients (Ghosh et al., 2015). In the next sections, we will review the important trafficking properties of HDL that are essential to its function.

## Mechanisms for Removal of Cellular Cholesterol

Cells store cholesterol, esterified to fatty acids, in the form of lipid droplets. Cells cannot catabolize cholesterol, and thus to prevent toxicity resulting from excessive accumulation, cellular free cholesterol from peripheral tissues must be removed (Yokoyama, 2005). One means of removing cholesterol from the bioactive pool is to form cholesteryl esters that are stored as lipid droplets inside of affected cells. The enzymes that esterify fatty acids to cholesterol are acyl-CoA: cholesterol acyltransferase 1 (ACAT1) and ACAT2 (Chang et al., 2009). Beyond esterification, free cholesterol can be physically removed from cells by passive (non-specific) diffusion or by apolipoprotein-mediated removal. Passive diffusion is a non-specific process between cell surface and extracellular acceptor, such as HDL. Passive diffusion of cholesterol can be enhanced via involvement of scavenger receptor B1 (SR-B1; Ji et al., 1997), and evidence demonstrates that ABC transporter G1 (ABCG1) can facilitate formation of mature HDL via passive diffusion (Kennedy et al., 2005). However, the more efficient cholesterol efflux mechanism is mediated through apolipoproteins. ABCA1, located on the plasma membrane (Wang et al., 2004), facilitates the generation of disk-like nascent HDL particles using cellular phospholipids, cholesterol, and lipid-poor ApoA1. While outside of the scope of this review, in depth discussion of the mechanisms of cellular cholesterol efflux has been expertly reviewed elsewhere (Phillips, 2014; Westerterp et al., 2014). Although HDL functions as a dedicated lipoprotein particle for accepting cholesterol, LDL, VLDL and liposomes may serve as alternative cholesterol acceptors (Tarling and Edwards, 2011). However, less LDL and VLDL passes into the interstitium as intact particles because they are larger than HDL (Michel et al., 2015).

## Reverse Cholesterol Transport and HDL Metabolism

HDL particles can be modified or removed from plasma in a variety of different ways. The major protein of HDL particles, ApoA1 (∼75%), is synthesized by the liver (70%) and also by the small intestine (30%). ApoA-II comprises about 20–25% of proteins in HDL. Nascent HDL particles produced from lipid-poor ApoA1 through ABCA1 expressed in hepatocytes and enterocytes can be further modified to form mature HDL through the enzyme lecithin cholesteryl acyltransferase (LCAT). LCAT esterifies cholesterol to form cholesteryl ester, the core of the mature particle. Another enzyme that is also able to modify HDL is the phospholipid transfer protein (PLTP). PLTP liberates phospholipid from apolipoprotein B (ApoB) containing lipoproteins like VLDL and LDL and transfers them to HDL, even as it also mediates HDL particle fusion to form larger HDL particles as well as some preβ-HDL. In PLTP^–/–^ mouse, HDL and ApoA1 levels are significantly decreased. The presence of preβ-HDL in PLTP^–/–^ mouse is much lower compared to the wild-type control (17% of that in wild-type; Jiang et al., 1999). HDL particles can be metabolized by the exchange of approximate equimolar of triglycerides with a cholesteryl ester to ApoB-containing lipoproteins, such as LDL and VLDL, by CETP in plasma (Tall, 1993). HDL can also be remodeled by various lipases, such as hepatic lipase or endothelial lipase, to form smaller HDL particles susceptible to faster catabolism. Finally, the major site of HDL cholesteryl ester uptake is the liver, accounting for 65% of the total, through HDL receptor, SR-B1 (Glass et al., 1983; Brunham and Hayden, 2015). The kidney accounts for 18% of total uptake of ApoA1 that contains <1% no cholesterol ester (Glass et al., 1983). Free ApoA1 is filtered through glomerular filtration and catabolized by proximal renal tubular epithelial cells. With all these mechanisms operating within plasma to regulate the composition and removal of HDL, it is easy to forget that the key role for HDL is to relocate from the plasma to the extravascular interstitium, where it can carry out its role in accepting cholesterol from cells in extravascular spaces.

## HDL Transport From Plasma to Lymph

**FIGURE 2:**
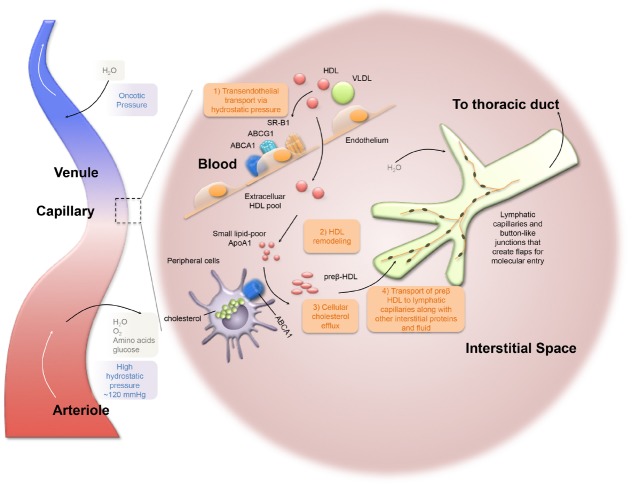
**Lymphatic dependent reverse cholesterol transport within the peripheral tissues.** HDL particles cross the vascular endothelium from plasma into interstitial fluid. Lipid-poor ApoA1 facilitates cellular cholesterol efflux through ABCA1-mediated pathway to form preβ-HDL. Lymphatic capillaries have discontinuous “button-like” junctions, which are permeable for optimal fluid uptake. Lymphatic flow is driven by the pumping action of downstream collecting lymphatic vessels (not depicted). Ultimately, lymph ends up in the thoracic duct that crosses the lymphovenous valve and drains into the subclavian vein.

While HDL is measured in the plasma, much of the life cycle of HDL is spent within the tissue (Rudra et al., 1984; Miller et al., 2013; Randolph and Miller, 2014) and its migration from that tissue back to the blood compartment occurs via trafficking through the lymphatic system. Radiolabeled cholesterol was identified in the lymph and tissue compartments immediately after i.v., administration in human patients (Reichl et al., 1973). Several weeks following administration, radiolabeled tissue cholesterol was higher in lymph than in plasma, indicating that cholesterol in lymph was derived from plasma (Samuel et al., 1972; Reichl et al., 1973). HDL particles have been proposed to cross the vascular endothelium from plasma into interstitial fluid through receptor-mediated transcytosis based on *in vitro* studies (Rohrer et al., 2006) and/or passive diffusion through intercellular pores (Nordestgaard and Nielsen, 1994; Michel et al., 2015; Figure 2). Using aortic endothelial cells *in vitro*, Cavelier et al. (2006) showed that lipid-free ApoA1 binds and is internalized and transported in an ABCA1 dependent manner, generating lipidated particles through this process. Also using aortic endothelial cells *in vitro*, Rohrer et al. (2009) demonstrated HDL particles bind, are internalized, and are transported in a SR-B1 and ABCG1, but not ABCA1, dependent process, reducing particle size without degrading the protein moiety. However, in rabbits fed a high cholesterol diet, the influx into the arterial space of plasma lipoproteins, such as LDL, VLDL, and HDL, decreases linearly with the logarithm of particle diameter (Stender and Zilversmit, 1981), suggesting that lipoproteins cross the endothelium in a size-dependent manner. Michel et al. (2015) demonstrated *in vivo* that HDL and LDL transport from the plasma to the interstitial space occurs passively through endothelial intercellular pores and that active receptor-mediated transcytosis is unnecessary. If transport is predominantly passive, particle size would be expected to limit the transfer rate. Indeed, studies indicate that an HDL size increase from average 4.5–6 nm lowers its clearance rate by 12% (Michel et al., 2015). When vascular permeability is increased, influx of HDL into interstitial fluid is enhanced, and the rate of RCT is increased. The result supports the observation of the importance of passive HDL transport (Kareinen et al., 2015). Adenoviral overexpression of PLTP in hepatocytes generates larger particle size (<7.1 nm but larger than lipid-free ApoA1; Ji et al., 2014) and also increases atherosclerotic lesion size in PLTP overexpressing ApoE deficient mice (Zhang et al., 2014), although the causal relationship between PLTP and CVD is still under debate (Vergeer et al., 2010; Kim et al., 2015). In line with the mechanism proposed by Zhang et al. (2014), the increased HDL particle size resulting from PLTP overexpression impaired RCT (Samyn et al., 2009), perhaps impairing movement of HDL into the interstitium, limiting cholesterol uptake from extravascular spaces like plaque, and ultimately increasing atherosclerotic disease progression. Whether increased HDL particle size affects HDL transport and/or RCT requires further investigation.

## The Lymphatic System and its Functions

Once in the interstitium, HDL picks up cellular cholesterol and, to complete its job, must return to plasma. HDL appears to return back to the plasma compartment via movement through the lymphatic network that has evolved to manage and prevent fluid accumulation in tissues. Starling (1896) proposed that fluid flux across the walls of blood capillaries was determined by differences in blood pressure and colloid osmotic pressure between the interstitial fluid and plasma. For example, on the arterial side of the capillary bed, where the blood pressure (∼120 mmHg) is higher than the colloid osmotic pressure, plasma constituents continuously filter into the interstitial space. On the venous side of the capillary bed, where the colloid osmotic pressure is higher than the blood pressure, some interstitial fluid may be reabsorbed as per Starling’s Law (Földi and Strössenreuther, 2005; Figure 2). However, it is now thought the venous system has a much more minor role than previously believed (Levick and Michel, 2010). Lymphatic capillaries, blind-ended vessels composed of a single layer of lymphatic endothelial cells (LECs) lacking a basement matrix, also drain fluid from the interstitial space. It is important to stress, however, that the lymphatics are not merely finishing the task carried out by the venous system, but the lymphatic vessels are able to take up and transport molecules (and cells) larger than those that will readily traverse the venous wall. Molecules larger in radius than tumor necrosis factor α (3.24 nm) are primarily transported via the lymphatic vasculature (Miller et al., 2011) and lipoproteins of course are much larger than this. Sorting of lipoproteins for transport based on size was also raised by the studies of Fraser et al. (2012) who argued that liver sinusoidal cells create a semipermeable barrier, “liver sieve,” to sort colloidal sized particles, prevent the uptake of chylomicrons and large VLDL, and permit the selective uptake of the remnant particles.

There is a structural basis for the ability of lymphatics to transport larger molecules than the venous vasculature. Unlike blood vessels, which have continuous inter-endothelial junctions, lymphatic capillaries have discontinuous “button-like” junctions, which are permeable for optimal fluid uptake (Baluk et al., 2007; Tammela and Alitalo, 2010; Figure 2). From the capillaries, the lymph flows into pre-collector lymphatic vessels and then into collecting lymphatic vessels. Collecting lymphatic vessels are very different from lymphatic capillaries in that they are surrounded by smooth muscle cells (SMCs) with intrinsic contractile activity that propels lymph (Muthuchamy and Zawieja, 2008) through units called lymphangions that are separated by bi-leaflet valves to prevent lymph backflow (Bazigou et al., 2009). Lymph propulsion is also influenced by external arterial pulsations, such as skeletal movements, interstitial pressure, and inflammatory responses, or by internal nitric oxide (NO) synthesis produced by endothelial nitric oxide synthase (eNOS) from LECs (Kesler et al., 2013). Ultimately, lymph is filtered through lymph nodes and the filtrate ends up in the thoracic duct that drains into the subclavian vein (Jeltsch et al., 2003). Lymphatic vessels exist in most tissues with the exception of the eye (cornea and retina), bone marrow, and brain parenchyma (Tammela and Alitalo, 2010). The brain does contain an elegant system that promotes very active fluid transport called the glymphatics (Iliff et al., 2013; Plog et al., 2015) and very recently lymphatics in the meninges were reported (Aspelund et al., 2015; Louveau et al., 2015) that are thought to receive fluid from the glymphatic system.

## HDL Transport During Reverse Cholesterol Transport Through Lymphatics

In the interstitial space, HDL becomes loaded with cellular cholesterol, as the first step of RCT, for transport back to the plasma compartment through the lymphatic system. Although the idea that HDL removal from tissues would follow osmotic flow of water into the lumen of venous capillaries (Perl, 1975) seems highly unlikely based on the principles of higher molecular weight molecules requiring lymphatics (Miller et al., 2011) and impressive recovery of HDL in human afferent lymph (Nanjee et al., 2000, 2001a,b; Miller et al., 2013), formal demonstration that lymphatics mediated transit of HDL from tissue back into blood has occurred only recently (Lim et al., 2013; Martel et al., 2013). Surgical interruption of tail lymphatics significantly reduced RCT, without altering the lipoprotein profile or macrophage cholesterol efflux capacity (Martel et al., 2013). These findings were replicated in a genetic mutant mouse model (Martel et al., 2013), which lack dermal lymphatic capillaries due to deficiency in one allele of vascular endothelial growth factor-C receptor (VEGFR3; Karkkainen et al., 2001; Martel et al., 2013). Furthermore, a surgical model of aortic arch transplant was used to argue that cholesterol removal from plaques was also dependent upon lymphatics (Martel et al., 2013). Models with lymphatic disruption, such as the aortic transplant or surgical disassociation, nicely demonstrate the necessity of lymphatics in RCT. However, to eliminate potentially confounding factors such as model inflammation, these mechanisms need to be investigated using non-surgical models.

## HDL Composition and Modification in Interstitial Space and in Lymph

Cholesterol concentration in the lymph is approximately a tenth of the concentration in the plasma (Reichl et al., 1978). As in the plasma, cholesterol in the lymph is transported by apolipoproteins, such as ApoA1 and ApoB, although Reichl and Pflug (1982) and Reichl et al. (1977) consistently found that ApoA1 and ApoB concentrations in the lymph were much lower than plasma concentrations (Sloop et al., 1983; Reichl et al., 1991; Randolph and Miller, 2014). During passage from the interstitial space to the lymph, Reichl et al. (1975) reported that plasma lipoproteins undergo modifications, demonstrated with radiolabeled apolipoproteins injected into male patients. In his studies, men were given an i.v., injection of ^131^I-labeled ApoB LDL, and ^125^I-labeled ApoA VLDL. Both ^131^I-labeled and ^125^I-labeled proteins appeared in the lymph. In lymph, ^125^I-labeled ApoA-containing lipoproteins showed similar mobility compared to ApoA1 of HDL in plasma, whereas ^131^I-labeled ApoB still remained with ApoB-containing lipoproteins, suggesting that ApoA-containing lipoproteins undergo modification during passage to the lymph (Reichl et al., 1975). The work of Reichl et al. (1975) provided the foundation for further study into lipoprotein remodeling within the interstitial fluid compared with plasma. Incubation studies found that preβ-HDLs were reduced in the first phase of plasma incubation (Sloop et al., 1983), due to the LCAT activity in plasma that esterifies free cholesterol of HDL facilitating conversion to mature spherical HDL (Sloop et al., 1983; Miller et al., 2013). Beyond 2 h of incubation in plasma, preβ-HDLs were recovered due to the further modification by PLTP and CETP, which facilitate the exchange of lipids with other lipoproteins (Sloop et al., 1983). In contrast, no reduction of preβ-HDLs were observed during incubation in human lymph samples: contributing factors include higher specific activity of PLTP, lower cholesterol esterification rate, and lower CETP activity than observed in plasma (Miller et al., 2013). Relevant to RCT, cholesterol removal from cells is most efficient with small, dense HDL particles (Du et al., 2015). These findings, together with previous observations (Castro and Fielding, 1988), suggest a feasible physiological mechanism within the interstitial space: increased conversion of preβ-HDLs from α-HDLs in the interstitial fluid makes more preβ-HDLs in lymph, thereby picking up available cholesterol from peripheral tissues/cells in an ABCA1-dependent process (Castro and Fielding, 1988), leading to the formation of discoidal HDLs (Figure 2).

Lymph apolipoprotein concentrations could vary depending on cholesterol feeding. The ratios of ApoE/ApoA1 and ApoAIV/ApoA1 in lymph HDL from control and cholesterol-fed dogs is increased by sevenfold compared to plasma HDL (Sloop et al., 1983). Upon discoidal HDLs entering the bloodstream via the thoracic duct, further modifications can occur by LCAT creating spheroidal α-HDLs with more cholesteryl esters in the core (Sloop et al., 1983). Spheroidal α-HDLs can be catabolized in the liver with direct uptake through HDL receptor, SR-B1, or CETP can facilitate transfer of cholesteryl esters to ApoB-containing lipoproteins, such as VLDL and LDL (Brunham and Hayden, 2015). Modification of HDL within the interstitial space and lymph may have a role in development of atherosclerosis, though a better understanding of these modifications that may be specific to the atherosclerotic plaque environment is needed.

## Lymphatic Significance and Implication for Cardiovascular Disease

**FIGURE 3:**
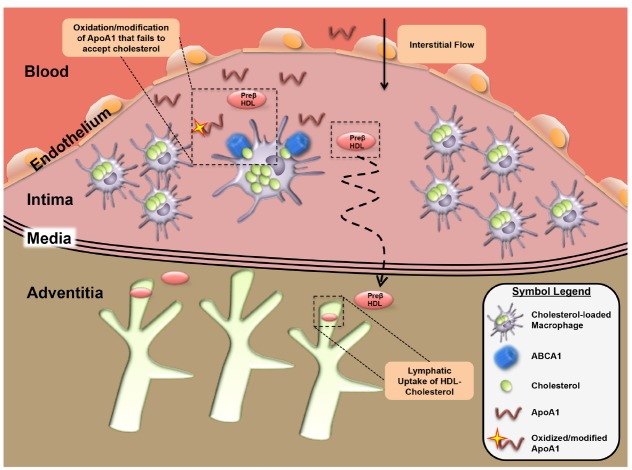
**Lymphatic dependent reverse cholesterol transport within the atherosclerotic plaque.** In the context of the atherosclerotic plaque environment, removal of plaque cholesterol may be impaired by oxidation and modification of ApoA1 that prevents its ability to accept cholesterol from foam cells to form HDL. Movement of the HDL-cholesterol out of plaques occurs in the direction of interstitial fluid flow with removal likely occurring in adventitial lymphatic vessels.

The influx, oxidization, and retention of ApoB-containing lipoproteins within the subendothelial space is a key aspect of the initiation of atherosclerosis (Tabas et al., 2007). Recruitment of monocytes, and differentiation into foamy macrophages, drive disease progression (Gautier et al., 2009). As macrophages phagocytize oxidized ApoB-containing lipoproteins, a fatty streak is formed within the intimal layer of the vessel, serving as a marker for the early stage of disease. Foam cells are thought to be inflammatory and necrosis of macrophages alongside invading smooth muscle cells with altered differentiation (Shankman et al., 2015) result in the formation of necrotic core and cholesterol crystals (Glass and Witztum, 2001). Is there a role for lymphatic vessels in plaque progression? A relationship between atherosclerosis and reduced lymphatic transport of cholesterol was hypothesized in the early 1980s (Lemole, 1981; Miller et al., 1992). Lymphatic vessels were later identified within the arterial wall (Johnson, 1969; Nakano et al., 2005; Drozdz et al., 2012). In human diseased vessels, a positive correlation exists between intimal thickness and adventitial lymphatic density (Drozdz et al., 2008), as well as lymphatic dysfunction and atherogenesis (Eliska et al., 2006). Perhaps these results can be interpreted as evidence that lymphatics remodel to facilitate RCT from the intimal environment. It remains unclear and imperfectly studied as to how HDL cholesterol leaves the plaque, although flow into lymphatics is experimentally supported (Martel et al., 2013) and most consistent with physiological principles (Figure 3). Specifically, Martel et al. (2013) transplanted plaque-laden aortic arches that were loaded with stable isotope-cholesterol into atherogenic recipient mice. Inhibition of the regrowth of lymphatic vessels, by neutralizing anti-VEGFR3 mAb treatment, impaired the efflux of cholesterol from the transplanted aortic plaque tissue. Further study is warranted to delineate the nature of the lymphatic system involvement in RCT from native plaque in a less manipulated system. We propose that HDL accepts cholesterol from plaque macrophages and then exits via the lymphatic network, and that very few macrophages themselves exit the plaque via lymphatics (Potteaux et al., 2011; Randolph, 2014; Figure 3). As mentioned earlier, to better clarify these mechanisms, models are needed that can avoid some caveats, one of which is the inherent inflammation of the surgical procedure that can promote neo-lymphangiogenesis (Aebischer et al., 2014).

One such model was utilized in a 2014 study by Vuorio et al. (2014). By crossing sVEGFR3 and Chy transgenic mice, which have insufficient lymphatics, with the atherogenic LDLR/ApoB48 deficient mice, Vuorio et al. (2014) demonstrated a deficiency in adventitial lymphatics of descending aorta. RCT rate from the peritoneum, measured by injection of radiolabeled-cholesterol macrophages in the peritoneum, was not different between sVEGFR3 transgenic x LDLR/ApoB48 and LDLR/ApoB48 deficient mice, although the peritoneal environment is a poor model for lymphatic transport, given the role of the omentum in uptake of fluid and cells in a manner seemingly independent of lymphatics (Yuan et al., 1994). Most importantly, these two lymphatic deficient transgenic strains had accelerated atherosclerosis and increased plasma cholesterol and triglyceride levels compared to lymphatic sufficient controls, indirectly supporting the role of lymphatic dependent mechanisms in the removal of cholesterol from the atherosclerotic vessel. What remains currently unknown is how modification of ApoA1 within the plaque environment, where dysfunctional HDL has been documented (Zheng et al., 2004; DiDonato et al., 2013; Huang et al., 2014), influences cholesterol transport from through the lymphatics.

In other studies, the cholesterol level of the skin has been correlated with the increased thickness of carotid intima area of subjects with (Bouissou et al., 1974, 1982) and without diagnosed CVD (Bouissou et al., 1974, 1982; Tzou et al., 2005; Lutgers et al., 2010). Therefore, skin cholesterol level is useful to identify potential individuals that may undergo atherosclerosis (Stein et al., 2008). Xanthoma, known as lipid and cholesterol accumulation in the skin, is induced by foam cell formation with accumulated lipoproteins, similar to the pathogenic mechanisms in the early stage of atherosclerosis (Rapp et al., 1983), and poor lymphatic drainage correlates to xanthoma formation in human patients (Berger et al., 1972). Relevant to atherosclerotic disease, deficiency of ApoE in human and mouse promotes development of xanthoma following prolonged hypercholesterolemia (van Ree et al., 1995; Feussner, 1996; Moghadasian et al., 2001). Lim et al. (2009) showed that hypercholesterolemic ApoE deficient mice, but not young ApoE-deficient mice, had structural and functional defects in skin lymphatics. These results suggest that the atherogenic phenotype of ApoE deficient mice may at least in part be due to defective lymphatic function that develops in response to hypercholesterolemia (Lim et al., 2009, 2013; Martel et al., 2013). Interestingly, mice deficient in both ApoA1 and LDLR have significantly increased atherosclerotic plaque burden and reduced RCT (Moore et al., 2003, 2005). A separate study found that despite a lack of plasma hypercholesterolemia, ApoA1/LDLR double deficient mice had substantial cholesterol accumulation in the skin (Zabalawi et al., 2007). This pathological change could be normalized by ApoA1 treatment (Wilhelm et al., 2010) or ectopic macrophage ApoA1 expression (Tavori et al., 2015), probably due to restored cellular cholesterol efflux out of the skin.

## Conclusion

Over the past 30 years, although observational studies suggest a hypothesis that higher HDL cholesterol would reduce cardiovascular events, recent data indicate a system more subtle and complex than that basic notion. It is widely recognized that what really matters is cholesterol flux and removal from cells like macrophages. However, relatively little research examines the trafficking of HDL into and out of the interstitium. As trafficking out of the interstitium appears to be dependent upon functional lymphatics, we suggest that additional research into the maintenance of lymphatic transport with respect to HDL trafficking is needed.

### Conflict of Interest Statement

The authors declare that the research was conducted in the absence of any commercial or financial relationships that could be construed as a potential conflict of interest.
